# Flapless Endoscopic Myringoplasty in Large Perforations: Is Tympanomeatal Flap Elevation Necessary?

**DOI:** 10.7759/cureus.96965

**Published:** 2025-11-16

**Authors:** Ismail Nakkabi

**Affiliations:** 1 ENT (Ear, Nose, Throat) Department, Oued Eddahab Military Hospital, Faculty of Medicine and Pharmacy of Agadir, Ibn Zohr University, Agadir, MAR

**Keywords:** cartilage graft, case report, endoscopic ear surgery, large tympanic membrane perforation, myringoplasty, tympanomeatal flap

## Abstract

The role of tympanomeatal flap elevation in type I tympanoplasty remains debated, particularly in cases of large tympanic membrane perforations. We report the case of a 59-year-old patient with a long-standing right-sided otorrhea and hearing loss. Otoscopic examination revealed a large inferior perforation involving the anteroinferior and posteroinferior quadrants. Audiometry demonstrated a 15 dB air-bone gap. The patient underwent flapless endoscopic myringoplasty using a 30° 4K endoscope. A bean-shaped cartilage with attached perichondrium was sculpted and positioned beneath the malleus handle, while a second perichondrial fragment was placed above the cartilage and beneath the tympanic membrane remnant, creating a sandwich configuration around the handle. The graft was stabilized with Gelfoam® (Pfizer Inc., New York, NY, USA), without elevation of the tympanomeatal flap. At six weeks, the graft was fully integrated with neovascularization, and the air-bone gap was reduced to 5 dB. These anatomical and functional outcomes were maintained at six months of follow-up. This case illustrates that flapless endoscopic myringoplasty using a cartilage-perichondrium graft can achieve stable anatomical and functional results in large perforations, while tympanomeatal flap elevation should be reserved for cases with suspected ossicular pathology or diagnostic uncertainty.

## Introduction

Tympanic membrane perforations are a common sequela of chronic otitis media and are frequently associated with hearing loss and recurrent infections. Surgical closure is indicated to restore the integrity of the tympanic membrane and improve hearing. Traditionally, type I tympanoplasty is performed with elevation of the tympanomeatal flap, which provides wide exposure of the middle ear but may increase operative time and morbidity [1]. However, conventional tympanomeatal flap elevation may also carry risks such as canal wall trauma, postoperative discomfort, and potential retraction of the reconstructed tympanic membrane due to flap manipulation.

With the advent of endoscopic ear surgery, less invasive approaches have been developed. Flapless endoscopic myringoplasty, performed without tympanomeatal flap elevation, allows direct transcanal access and placement of perichondrium or cartilage-perichondrium grafts. Comparative studies and meta-analyses have shown that endoscopic techniques provide outcomes comparable to conventional microscopic tympanoplasty, while offering advantages such as shorter operative times and reduced morbidity [1,2]. However, in cases of large perforations, the need for tympanomeatal flap elevation remains debated. The present report addresses this issue through the description of a case of flapless endoscopic myringoplasty for a large perforation.

## Case presentation

A 59-year-old patient presented with a long-standing history of right-sided otorrhea and hearing loss. Otoscopic examination revealed a large inferior tympanic membrane perforation, involving both the anteroinferior and posteroinferior quadrants, corresponding approximately to 70%-80% of the tympanic membrane surface, with well-defined margins and no active infection. The ossicular chain was not visible through the perforation. There were no signs of cholesteatoma or middle ear granulation tissue.

Audiometric evaluation demonstrated a conductive hearing loss with an air-bone gap averaging 15 dB. The contralateral ear was normal. The patient had no significant comorbidities, and surgical repair of the perforation was indicated.

The intervention was performed under general anesthesia using a 30° Karl Storz rigid endoscope connected to a 4K Storz camera system, via a transcanal approach, without tympanomeatal flap elevation.

Operative steps

**Figure 1:**
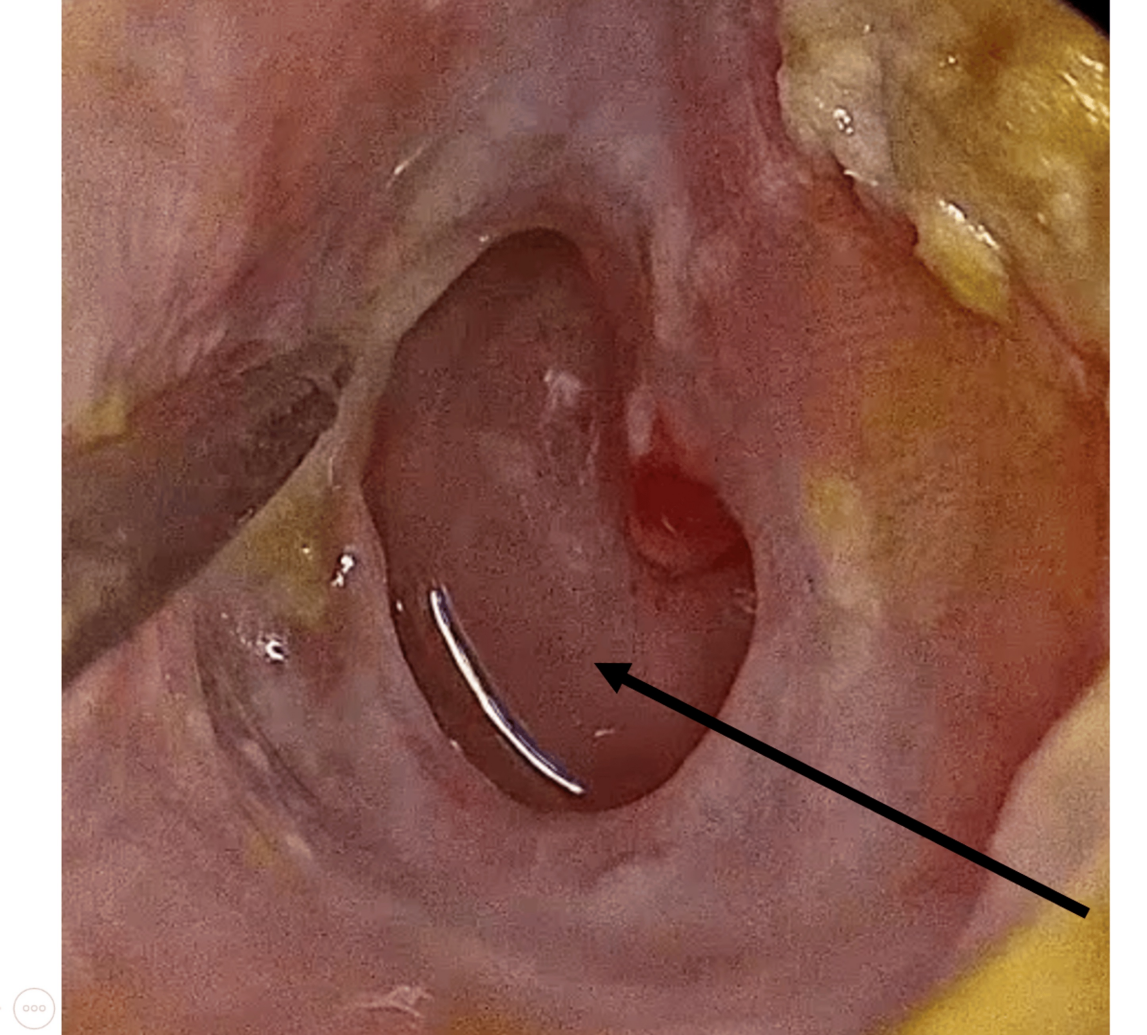
Large inferior tympanic membrane perforation (black arrow) involving the anteroinferior and posteroinferior quadrants.

The large inferior perforation can be visualized, confirming its extension across the anteroinferior and posteroinferior quadrants (Figure 1).

**Figure 2:**
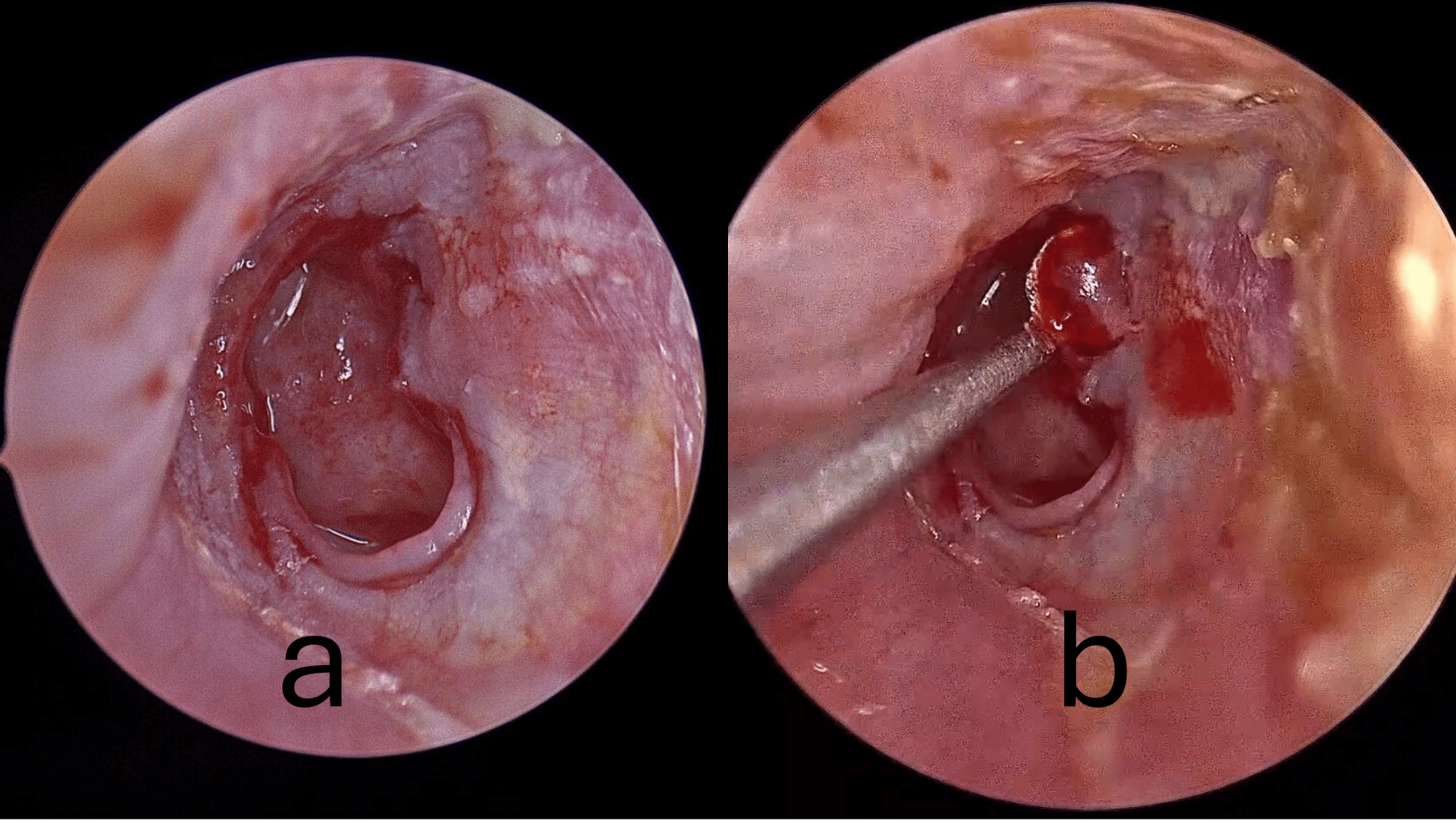
Skeletonization of the malleus handle (b) after refreshing of the perforation margins (a).

*Refreshing of perforation margins*: epithelial borders were excised to expose vascularized edges and optimize graft uptake (Figure 2a). 

*Skeletonization of the malleus handle*: After margin preparation, mucosal and fibrous adhesions were dissected, leaving the osseous surface of the handle exposed (Figure 2b).

**Figure 3:**
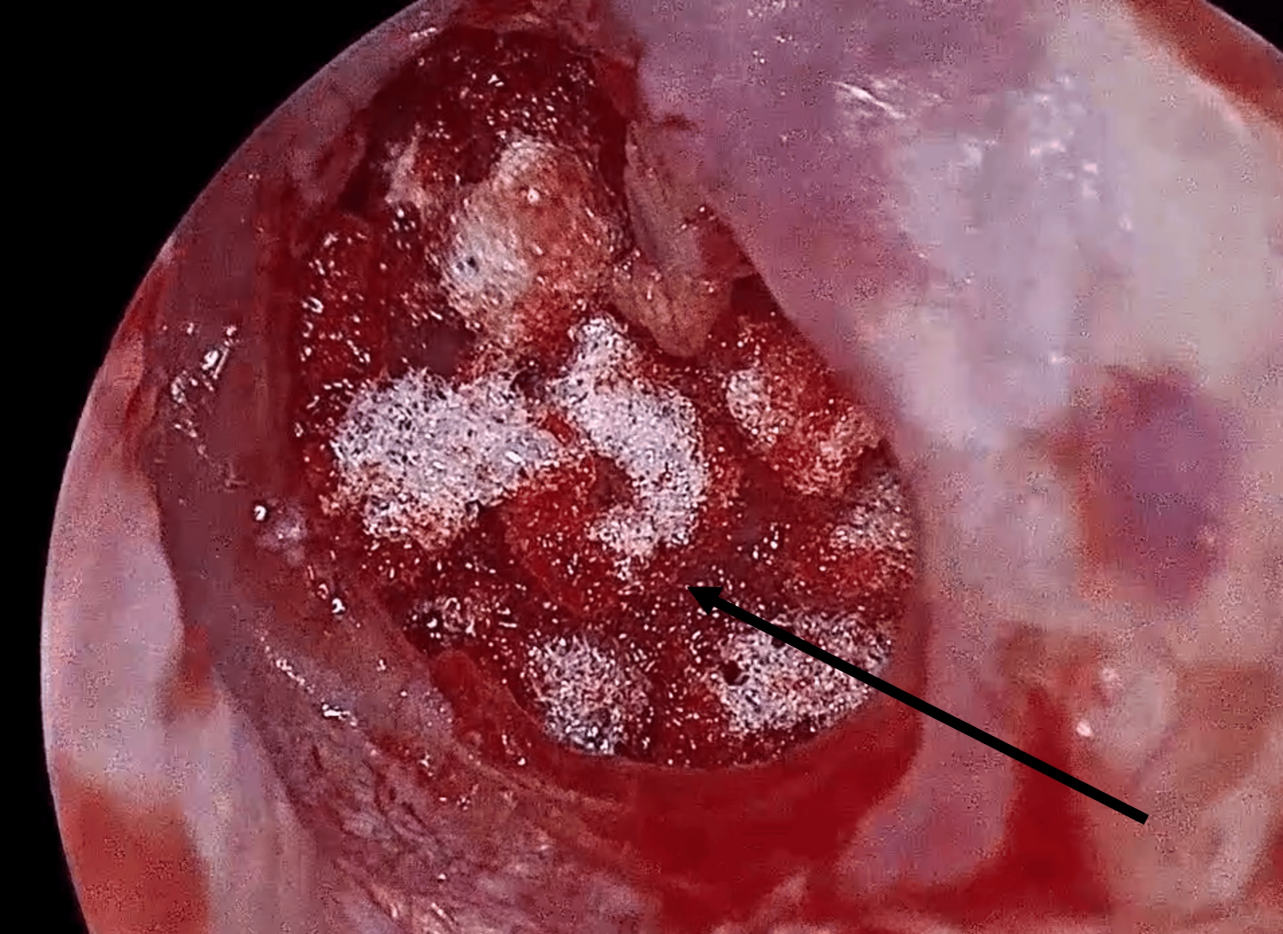
Middle ear cavity filled with absorbable gelatin sponge (Gelfoam) for graft support (black arrow).

*Middle ear preparation*: The tympanic cavity was partially filled with absorbable gelatin sponge (Gelfoam®, Pfizer Inc., New York, NY, USA) to provide a stable bed for graft placement (Figure 3).

*Graft harvesting and preparation*: Tragal cartilage with perichondrium was harvested. The cartilage was sculpted into a bean-shaped fragment with a preserved perichondrial rim.

*First layer placement*: The perichondrial extension of the cartilage was positioned beneath the malleus handle in underlay fashion.

**Figure 4:**
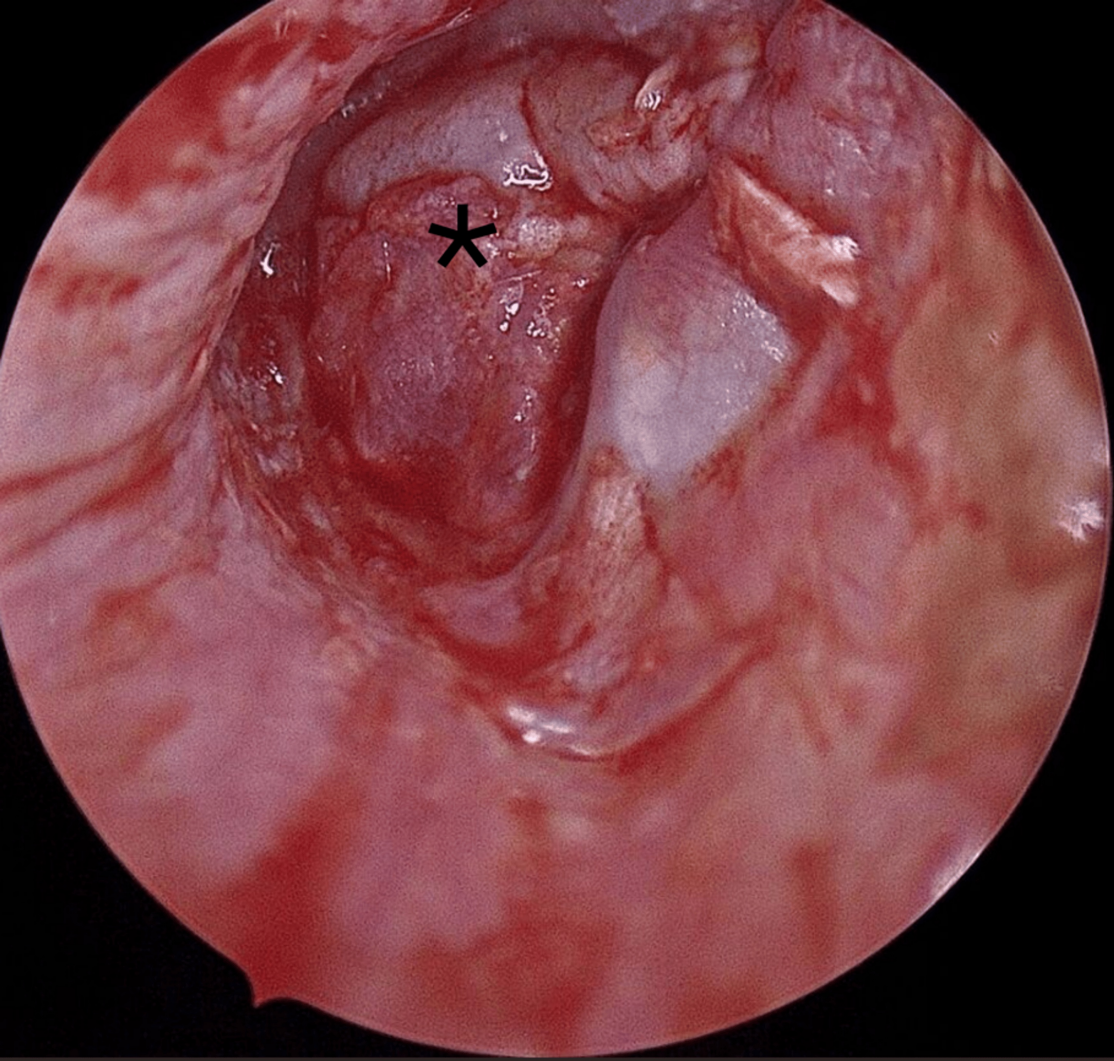
Bean-shaped cartilage with its perichondrium positioned beneath the malleus handle, and a second perichondrial fragment placed above it, creating a sandwich configuration (asterix).

*Second layer placement*: A separate perichondrial fragment was prepared and placed above the cartilage and above the malleus handle, thus sandwiching it. This second perichondrial sheet was inserted beneath the residual tympanic membrane to ensure full closure (Figure 4).

*Final stabilization*: Additional Gelfoam was placed lateral to the graft. No graft material was positioned lateral to the tympanic membrane remnant.

Postoperative outcome

**Figure 5:**
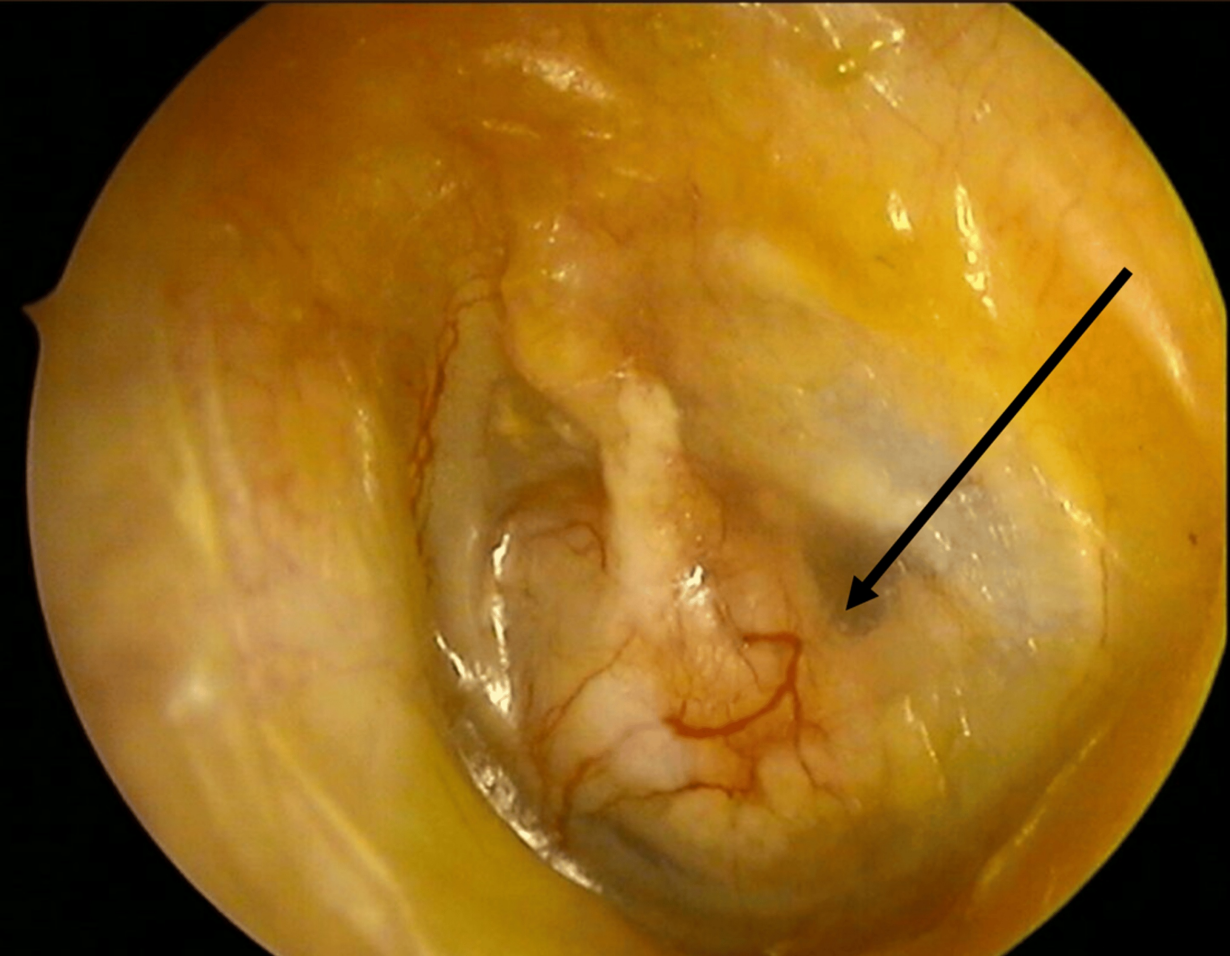
Postoperative view at six weeks showing graft integration with neovascularization (black arrow), indicating successful healing.

The reconstruction appeared stable, with the malleus securely encased between the cartilage-perichondrial layers. At six weeks, otoscopic examination showed a well-integrated graft with neovascularization (Figure 5), confirming biological uptake and healing. The audiogram demonstrated closure of the air-bone gap from 15 dB preoperatively to 5 dB postoperatively, indicating excellent functional recovery. These anatomical and functional results were maintained at six months of follow-up, with a stable graft and preserved hearing improvement.

Didactic video

A didactic operative video (Video 1) accompanies this report, demonstrating the entire procedure: visualization with a 30° 4K endoscope, refreshing of perforation margins, skeletonization of the malleus handle, preparation of the bean-shaped cartilage graft, dual perichondrial sandwich configuration, and postoperative assessment. Additional details regarding graft shape and handling are provided in the accompanying surgical video.

**Video 1:** Flapless endoscopic myringoplasty Flapless endoscopic myringoplasty in a large inferior perforation using a bean-shaped cartilage-perichondrium graft with sandwich configuration around the malleus handle.

## Discussion

The choice between flapless and flap elevation techniques in myringoplasty remains a matter of debate, particularly in the context of large tympanic membrane perforations. In our case, we achieved a stable anatomical and functional result using a flapless endoscopic approach with a cartilage-perichondrium composite graft, which emphasizes the feasibility of this technique in selected patients. From a biomechanical standpoint, the sandwich configuration provides enhanced structural stability and resistance to retraction. The dual perichondrial layers surrounding the cartilage improve graft integration by maintaining contact with well-vascularized surfaces on both sides, thus ensuring better neovascularization and long-term durability.

Several studies have compared the two approaches. Alzoubi et al. [3] conducted a prospective randomized trial and reported that both transtympanic (flapless) and tympanomeatal flap elevation techniques yielded comparable closure and hearing results overall, although the flap elevation technique tended to perform better in large perforations. This finding is particularly relevant to our case, as it underscores the importance of careful patient selection when considering flapless techniques for extensive defects.

More recently, Wang and Wang [4] highlighted the advantages of flapless endoscopic myringoplasty, especially in reducing surgical time, morbidity, and postoperative discomfort, while maintaining graft success rates comparable to conventional techniques. These observations support the idea that flapless techniques can be safely applied by experienced hands, with particular benefits in minimally invasive otologic surgery.

Kaya et al. [5] evaluated a modified technique using a limited tympanomeatal flap incision during endoscopic cartilage tympanoplasty. They reported a 100% graft take rate and significant hearing improvement across frequencies, suggesting that smaller or more selective incisions may preserve vascular supply and minimize trauma, thereby optimizing graft healing. This intermediate approach may combine the benefits of full flap elevation (exposure of the ossicular chain when required) with the reduced morbidity of flapless surgery.

Other authors have emphasized the role of cartilage-perichondrium grafts in providing stability, particularly in large or anterior perforations. Dündar et al. [6] and Ayache [7] demonstrated that chondroperichondrial techniques can effectively prevent lateralization and improve long-term graft uptake, while Chatelet et al. [8] confirmed the safety and feasibility of flapless approaches even in pediatric populations. Comparative analyses and meta-analyses have further confirmed that endoscopic tympanoplasty offers outcomes comparable to conventional microscopic techniques, with reduced morbidity [1,2].

Taken together, these studies demonstrate that flapless endoscopic myringoplasty can achieve excellent outcomes in terms of perforation closure and hearing gain, particularly when cartilage-based grafts are employed. However, for large perforations or when ossicular pathology is suspected, tympanomeatal flap elevation remains valuable because it allows direct inspection and potential intervention on the ossicular chain. Therefore, surgical decision-making should be individualized: flapless approaches may be favored in straightforward cases with intact ossicular chains and well-visualized perforation margins, while flap elevation should not be hesitated in cases of audiometric air-bone gap widening or suspicion of ossicular pathology.

Our case adds to the growing body of evidence suggesting that, when carefully selected, flapless endoscopic myringoplasty is a reliable technique even for large perforations, provided that stable grafting techniques such as cartilage-perichondrium composites are employed.

The durability of the result in our patient, maintained at six months, further reinforces the reliability of this approach in selected cases. Table 1 summarizes the advantages and drawbacks of performing myringoplasty with versus without tympanomeatal flap elevation.

**Table 1:** Advantages and limitations of myringoplasty with versus without tympanomeatal flap elevation

Aspect	Flapless Endoscopic Myringoplasty	With Tympanomeatal Flap Elevation
Surgical exposure	Limited visualization of ossicular chain [3]	Full exposure of the middle ear; ossicular inspection possible [3]
Operative time	Shorter, less invasive [2]	Longer due to flap elevation and repositioning [2]
Postoperative morbidity	Reduced canal trauma, less pain, and faster recovery [2]	Slightly higher morbidity due to flap handling [2]
Anatomical success (large perforations)	High closure rates with cartilage-perichondrium grafts [4]	Reliable, depends on flap healing and graft stability [4]
Functional outcomes	Comparable hearing improvement in selected cases [5]	Good results when ossicular exploration is required [5]

## Conclusions

Flapless endoscopic myringoplasty using a composite cartilage-perichondrium graft anchored to the malleus can achieve reliable closure and functional improvement even in large tympanic membrane perforations. This minimally invasive approach reduces morbidity and operative time while providing stable anatomical outcomes, with results maintained at six months of follow-up. Nevertheless, tympanomeatal flap elevation should not be disregarded when audiometric findings suggest an ossicular gap or when middle ear pathology is suspected, as it remains indispensable for diagnostic assessment and therapeutic intervention. Surgical strategy must therefore be tailored to the patient’s perforation size, location, and preoperative audiological profile.
